# Applications of generative AI in peer review process: friend or foe?

**DOI:** 10.1097/JS9.0000000000001689

**Published:** 2024-05-22

**Authors:** Haiyang Wu, Zaijie Sun, Qiang Guo, Cheng Li

**Affiliations:** a Department of Orthopaedics, The First Affiliated Hospital of Zhengzhou University, Zhengzhou; b Department of Clinical College of Neurology, Neurosurgery and Neurorehabilitation, Tianjin Medical University; c Department of Orthopedics, Baodi Clinical College of Tianjin Medical University, Tianjin; d Department of Orthopaedic Surgery, Xiangyang Central Hospital, Affiliated Hospital of Hubei University of Arts and Science, Xiangyang; e Department of Spine Surgery, Wangjing Hospital, China Academy of Chinese Medical Sciences, Beijing, China; f Center for Musculoskeletal Surgery (CMSC), Charité-Universitätsmedizin Berlin, Corporate Member of Freie Universität Berlin, Humboldt University of Berlin, Berlin Institute of Health, Berlin, Germany

*Dear Editor,*

In recent years, the rapid advancement of generative artificial intelligence (AI) has profoundly impacted various fields. According to our rough estimate, the *International Journal of Surgery* has published more than 80 papers related to generative AI^1,2^. Recent studies have indicated generative AI, such as ChatGPT, has permeated the realm of the peer review process, although the specific applications remain elusive^3,4^. Liang *et al*.^5^ analyzed alterations in peer review content across multiple conferences before and after the introduction of ChatGPT. The findings reveal a notable increase in the proportion of peer review texts within conferences that were either generated by or substantially modified by generative AI following the release of ChatGPT.

Generative AI has outstanding auxiliary advantages in peer review in many aspects, including language and formatting checks, plagiarism detection, and even matching potential reviewers. Its efficiency and accuracy are expected to improve the efficiency and quality of peer review, bringing a faster and more reliable review experience to the academic community. However, with this comes a series of controversial issues, especially regarding how to maximize the use of generative AI to improve academic productivity while maintaining academic rigor. This balance must be carefully considered when journal editors consider adopting these AI tools.

First of all, the use of generative AI requires strict compliance with the principles of transparency and traceability. Even if AI systems could produce content that appears to meet academic standards, academic journals should ensure that the generated content can be reviewed, traced back to its source, and presented transparently to peer review experts. To this end, it is crucial to develop a standardized set of metadata and review processes.

Secondly, when academic journals consider using generative AI, they should prioritize its role as an auxiliary tool rather than completely replacing human peer review. The intuition and judgment of human experts are critical in ensuring the quality of scholarship and the accuracy of the review process. Generative AI is able to provide rapid initial screening and draft writing, but final review and decision-making should still be made by human experts.

Most importantly, the academic community needs to work together to develop specific guidelines and standards to regulate the use of generative AI in peer review. These guidelines should cover aspects such as data use and privacy protection, algorithm transparency, copyright, and intellectual property rights. By developing unified guidelines, we could ensure that different journals and academic institutions follow similar standards when using generative AI, thus increasing the transparency and credibility of the entire peer review system.

In summary, skillfully integrating generative AI technology into peer review undoubtedly enhances the efficiency of scholarly publication. The key to harnessing this power lies in our adept utilization of generative AI as a tool for assisting peer review, ensuring generative AI technology serves our creativity rather than constrains our thinking. Therefore, we urgently need to build a consensus in the academic community and formulate clear guidelines for generative AI in peer review to ensure that it improves academic productivity without compromising the rigor and credibility of academic publishing.

## Ethical approval

This study does not include any individual-level data and thus does not require any ethical approval.

## Sources of funding

This study is supported by China Postdoctoral Science Foundation (2022M720385) and Beijing JST Research Funding (YGQ-202313).

## Author contribution

H.W.: conceptualization, methodology, data curation, formal analysis, resources, investigation, and writing – original draft; Z.S. and Q.G.: conceptualization, methodology, data curation, formal analysis, resources, and investigation; C.L.: methodology, data curation, formal analysis, resources, investigation, and writing – review and editing.

## Conflicts of interest disclosure

The authors declare no conflicts of interest.

## Research registration unique identifying number (UIN)

1. Name of the registry: not applicable.
2. Unique identifying number or registration ID: not applicable.
3. Hyperlink to your specific registration (must be publicly accessible and will be checked): not applicable.

## Guarantor

Haiyang Wu and Cheng Li.

## Data availability statement

The data underlying this article will be shared by the corresponding author on reasonable request.
